# An Improved Grain Growth Model and Its Application in Gradient Heat Treatment of Aero-Engine Turbine Discs

**DOI:** 10.3390/ma16196584

**Published:** 2023-10-06

**Authors:** Zhaofeng Liu, Chao Wang, Junyi Cheng, Jianzheng Guo

**Affiliations:** 1 State Key Laboratory of Powder Metallurgy, Central South University, Changsha 410083, China; liuzhaofeng@csu.edu.cn (Z.L.); cjunyi@hotmail.com (J.C.); 2 Shenzhen Wedge Central South Research Institute Co., Ltd., Shenzhen 518035, China

**Keywords:** dual microstructure turbine disk, finite element, grain growth, heat treatment, high-temperature alloy

## Abstract

A new grain growth model was developed by introducing the ultimate grain size to the traditional model. The grain growth behavior and its ultimate size under the Zenner pinning force are also discussed. This model was applied to the nickel-based superalloy and integrated into an FEM code. The grain evolution of a forged third-generation powder superalloy heat treated at different temperatures and holding times was studied. A gradient heat treatment setup was designed and implemented for a full-size turbine disc based on the model prediction to meet the accurate dual-microstructure requirements of an advanced aero-engine turbine disc design. The predicted temperature was validated by thermal couple measurements. The relative error between the prediction and the measurements is less than 2%. The metallographic examination of the whole turbine disk through sectioning showed that the grain size was ASTM 7-8 at the rim area and ASTM 11-12 at the bore region, which agrees well with the prediction. The predicted values of the three measurement areas are ASTM 12.1, ASTM 9.1, and ASTM 7.1, respectively, with a maximum error of 5% compared to the measured values. The proposed model was validated and successfully applied to help manufacture a dual-microstructure aero-engine turbine disc.

## 1. Introduction

Grain size is a very important factor in determining the properties of polycrystalline materials, such as tensile and creep properties. Hence, it is critical to understand and further predict grain growth during manufacturing processing. There are several ways to predict grain growth, such as phase field and cellular automata, which mainly focus on the change of microstructure and morphology in the process of grain growth at the microscale [1,2]. Due to its low computational efficiency, it is normally hard to apply to large structural parts. The deterministic kinetics model for grain growth is widely used in the prediction of grain size with high efficiency and reasonable accuracy.

A simple model for isothermal grain growth was proposed by Beck, which can be expressed as [3]:

(1) $$D=Kt^{\frac{1}{n}},$$

By introducing an initial grain size $D_{0}$, it can be rewritten as:

(2) $$D-D_{0}=Kt^{\frac{1}{n}},$$

where D,  t, and n are grain size, annealing time, and time-dependent exponent, respectively. The parameter K is a temperature-dependent constant and can be calculated as:

(3) $$K=K_{0}\exp\left(-\frac{Q}{RT}\right),$$

where $K_{0}$, Q, R, and T are a constant, grain growth activation energy, gas constant, and annealing temperature, respectively. Burke and Turnbull [4] proposed the grain growth exponent n=2 by combining the K factor with the surface energy of the boundary and the grain atomic volume. The kinetics of austenite grain growth under isothermal conditions is frequently explained by the Arrhenius constitutive relationship, which is based on a thermally activated atomic movement process. Sellars and Whiteman [5] suggested:

(4) $$D^{n}-D_{0}^{n}=Atexp\left(-\frac{Q}{RT}\right),$$

where A is the generalized mobility constant, a large value of n up to 10 was utilized. Du [6] suggested that a holding time exponent m should not be ignored and proposed an equation of:

(5) $$D^{n}-D_{0}^{n}=At^{m}\exp\left(-\frac{Q}{RT}\right),$$

Based on the above models, researchers have conducted a lot of valuable work. Zhong et al. [7] fitted the grain growth equation of oil well steel pipe under different insulation temperatures based on the Beck empirical formula and applied it to industrial production. Tian G et al. [8] fitted the kinetic model through isothermal grain coarsening tests and applied it to the heat treatment of powder turbine disks to predict the grain size. More studies have been conducted on the values of n and Q in the kinetic model under different materials and temperatures, in order to obtain better prediction results [9,10,11,12]. Unfortunately, most models above are limited to grain growth at constant temperature and cannot overcome the drawback that the grain grows continuously with time based on the model calculation.

Due to the formation of a second phase and impurities, the grain can normally only grow to an ultimate size for most alloys. The main driving force behind grain growth is the grain boundary decreasing due to the grain size increasing in polycrystalline metal materials. There is no inherent relationship between the grain size and the particle pinning characteristics of polycrystalline metals, such as nickel-based powder metallurgy superalloys which normally contain precipitate-strengthening particles [13]. The particle size and its distribution may remain relatively unchanged, regardless of the size of the matrix grains. However, the size and volume fraction of the precipitation particles located at grain boundaries play a crucial role in limiting grain boundary migration and affecting the kinetics of grain growth. As the heat treatment temperature and holding time increase, the precipitates experience a decrease in volume fraction and an increase in size. As such, the Zenner pinning force generated by these particles decreases in accordance with the following equation [14]:

(6) $$F=C\gamma \frac{f}{r},$$

where C is a constant, γ represents the grain boundary energy, f and r denote the volume fraction and radius of undissolved coarse particles, respectively. For nickel-based powder metallurgy superalloys, with the heat treatment temperature increasing, the volume fraction of γ' phase at the grain boundary decreases. As a result, the grain boundary is pinned less effectively. With prolonged holding time at elevated temperature, according to the mechanism of Oswald ripening, the radius of γ' phase at the grain boundary increases with the dissolution of small particles, or by the mechanism of precipitate agglomeration [15], the γ' phase at the grain boundary aggregates and connects, which also reduces the pinning force. Thus, the particle pinning forces determine the final grain size more effectively than the driving forces.

In polycrystalline alloys, grain boundaries refer to the interfaces between individual grains. The presence of these boundaries increases the overall free energy of the alloy. During heat treatment, grain boundary energy decreases as grain size increases, promoting grain growth. Crystalline thermodynamic stability also contributes to such growth, as smaller grains tend to be less stable thermodynamically. Thus, increasing grain size reduces the instability and allows the crystal coarsening to reach a more stable state. During sub-solvus heat treatment, grain growth stops when precipitation pinning forces at the grain boundary are stronger than the driving force for grain growth. Even for heat treatment at super-solvus temperature without precipitates, grains will not grow forever [16]. An ultimate grain size threshold exists, which should be incorporated into the grain growth model.

To consider the Zener pinning effect in the grain growth model, an improved one was proposed after modifying Equation (4) by introducing a limit grain size term and integrating it into an FEM code. The code was applied to simulate the heat treatment process of a turbine disc made with a third-generation nickel-based powder superalloy, FGH4113A [17,18]. The model parameters were calibrated through isothermal grain coarsening experimental results. The proposed model was validated and successfully applied to help manufacture a dual-microstructure aero-engine turbine disc.

## 2. Grain Growth Model Development

If the Zener pinning effect were considered, the rate of growth would decrease to zero as the grain size approached the ultimate size. To simplify the following mathematical derivation, the exponential grain size $D^{n}$ is treated as a variable, and Equation (4) can be rewritten in a differential form:

(7) $$∆\left(D^{n}\right)=K∆t,$$

If the ultimate grain size $D_{ult}^{n}$ is known, instead of constant grain growth rate ($\frac{∆(D^{n})}{∆t}$), it is assumed that the growth rate is linearly related to the difference between the ultimate size and the current size. Then the incremental form of the new equation is:

(8) $$∆\left(D^{n}\right)=\left(D_{ult}^{n}-D^{n}\right)K_{ult}∆t,$$

If the monotonicity of grain growth is considered:

(9) $$∆\left(D^{n}\right)=\max\left(D_{ult}^{n}-D^{n},0\right)K_{ult}∆t,$$

Equation (9) can be applied to calculate the grain growth during the continuous heating process. In order to obtain the model parameters based on isothermal grain growth experiments, the proposed model can be expressed in an integral form:

(10) $$D^{n}=D_{ult}^{n}+\left(D_{0}^{n}-D_{ult}^{n}\right)\exp\left(-K_{ult}t\right),$$

Like the original model, the parameter $K_{ult}$ at different temperatures also can be expressed as:

(11) $$K_{ult}=A_{ult}\exp\left(\frac{-Q_{ult}}{RT}\right),$$

**Figure 1:**
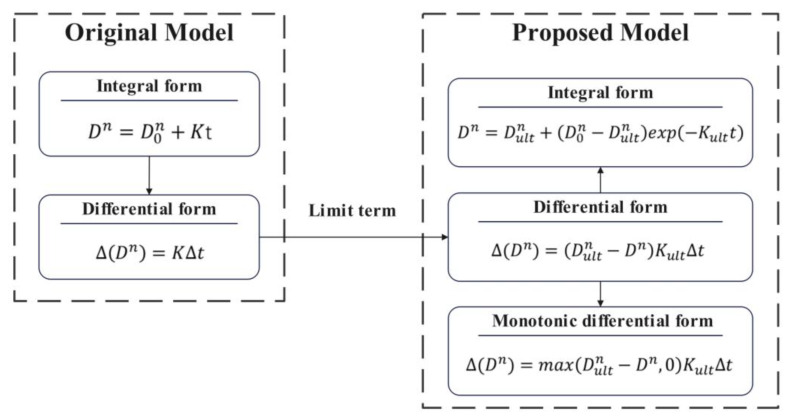
Derivation process of the model.

The derivation process of the model is shown in Figure 1.

The proposed model, based on the Zener pinning effect, describes how the grain size approaches the ultimate size. The growth rate slows down due to the increasing influence of pinning obstacles. To simplify the problem, the proposed grain growth rate is derived from the original model by considering the linear influence of the difference between the ultimate size and the current size. The incremental form of equation (Equation (8)) can be rewritten as follows:

(12) $$∆\left(D^{n}\right)=\frac{\left(D_{ult}^{n}-D^{n}\right)}{D_{ult}^{n}}\left(D_{ult}^{n}K_{ult}\right)∆t,$$

If $D_{ult}^{n}\gg D^{n}$, $\frac{\left(D_{ult}^{n}-D^{n}\right)}{D_{ult}^{m}}\approx 1$, then:

(13) $$∆\left(D^{n}\right)\approx \left(D_{ult}^{n}K_{ult}\right)∆t,D_{ult}^{n}\gg D^{n},$$

This equation has the same form to the original model (Equation (7)), when:

(14) $$K=D_{ult}^{n}K_{ult},$$

when the ultimate size is much larger than the grain size, it will have little influence on the grain growth rate. Let $K_{ult}=\frac{K}{D_{ult}^{n}}$, Equations (8) and (10) can be expressed by K that is from the original model as follows:

(15) $$∆\left(D^{n}\right)=\frac{\left(D_{ult}^{n}-D^{n}\right)}{D_{ult}^{n}}K∆t,$$

(16) $$D^{n}=D_{ult}^{n}+\left(D_{0}^{n}-D_{ult}^{n}\right)\exp\left(-\frac{K}{D_{ult}^{n}}t\right),$$

**Figure 2:**
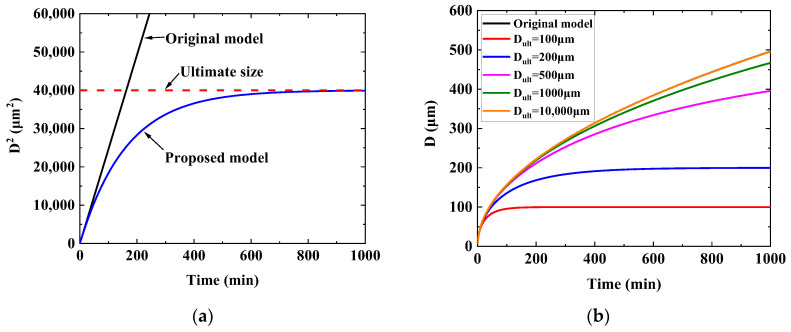
Comparisons of the original model and the proposed model. (**a**) Different models approaching the ultimate size; (**b**) the influences of different ultimate grain sizes.

Based on Equations (10) and (16), Figure 2 compares the two models by setting the same K value. Figure 2a shows how the grain size deviates from the original model to the current proposed one. Figure 2b presents the influences of different ultimate grain sizes on grain growth. It is found that the curve of the proposed model approaches the original model if the ultimate size is large enough. The two curves overlap when $D_{ult}$ = 10,000 μm for this calculation.

**Figure 3:**
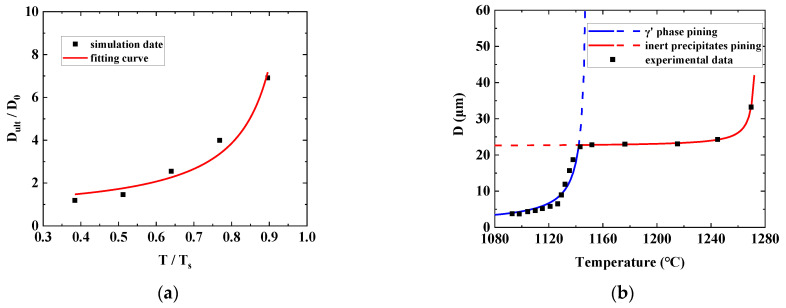
Ultimate grain size fitted by Equation (17). (**a**) The results of polycrystalline molecular dynamics simulation [19]; (**b**) experiment results under multiple pining phases [16].

According to Equation (6), the limit term $D_{ult}$ is affected by the grain boundary energy, the volume fraction, and the radius of the second phase. Many researchers have studied at the microscale, but these models are too complicated to be applied in heat treatment analysis. Since the influencing factors are all temperature dependent, the limit terms can be simplified to a function expressed in terms of temperature. It is proposed as follows:

(17) $$D_{ult}=\frac{a}{\left(1-{\left(\frac{T}{T_{s}}\right)}^{b}\right)},$$

where, a and b are material parameters, $T_{s}$ is the solvus temperature of the pining phase, T is the heat treatment temperature. Once the temperature exceeds $T_{s}$, the pining effect disappears, and the grain will grow continuously following the original model. E. A, Holm et al. [19] used large-scale polycrystalline molecular dynamics simulation to study the influence mechanism of the smooth grain boundary on grain size. The simulation results can be plotted by Equation (17) with parameter fitting, as shown in Figure 3a. Song et al. [16] studied the ultimate grain size that was influenced by the multiple pinning effects of the second-phase particles. Below the γ’ phase solvus temperature, γ’ has a strong inhibitory effect. While beyond the solvus temperature, oxides and carbides play a dominant role. Equation (17) can be applied to each pining mechanism as well, and the fitting results are presented in Figure 3b.

## 3. Experimental Materials and Methods

In order to obtain the needed parameters of the equation, extensive isothermal grain growth experiments were designed and performed.

The alloy used in the experiment is the third-generation nickel-based powder superalloy, FGH4113A. Table 1 gives the nominal composition. The main process route of the alloy is: vacuum induction melting (VIM) + argon atomization (AA) + hot isostatic pressing (HIP) + hot extrusion (HEX) + isothermal forging (IF).

The master alloy ingot is melted in a vim-80II 500 kg vacuum induction melting furnace with a working vacuum of 10^−3^ Pa. The argon atomization powder production process adopts VIGA (100 kg) equipment. The powders were sieved to 270 mesh, vacuum degassed, filled into a container, sealed, and HIPped to a cylindrical part. The HIP process condition was to raise the temperature and pressure to 1150 °C and 150 MPa in 4 h, hold for 4 h, and then cool down with the furnace. Then a Φ105 × 1040 mm bar was extruded by a 5000 t horizontal extruder at an extrusion temperature of 1120 °C, an extrusion speed of 25 mm/s, and an extrusion ratio of 5:1. Finally, a Φ200 mm experimental disk blank was made by a 3000 t vertical die forging hydraulic press.

In order to establish the relationship between heat treatment temperature and grain size, extensive isothermal grain growth tests were performed with a KSL-1400 muffle furnace. The temperatures were set at 1060 °C, 1120 °C, 1160 °C, and 1180 °C, respectively, with different soaking times followed by air cooling as shown in Table 2. The grain sizes of the test pieces were measured to study the effect of heat treatment parameters. The intercept method was used to count the grain size grade of the test piece, and the measurement standard is in accordance with GB/T6394-2017 [20].

## 4. Experimental Results and Model Parameter Determination

### 4.1. Experimental Results

**Figure 4:**
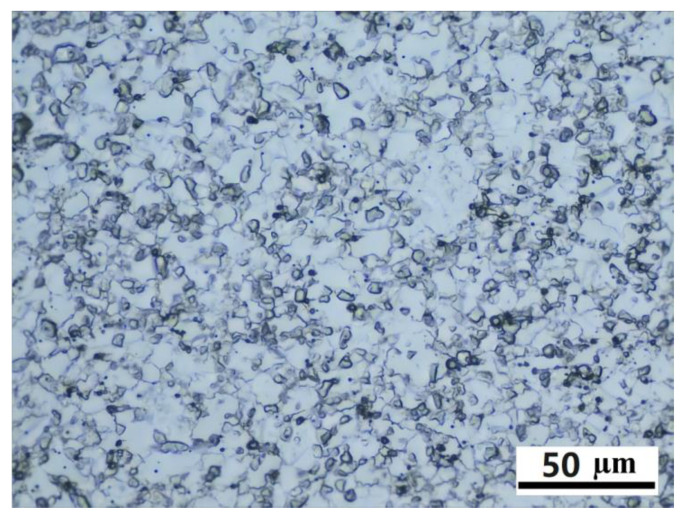
Initial-forged state microstructure.

**Figure 5:**
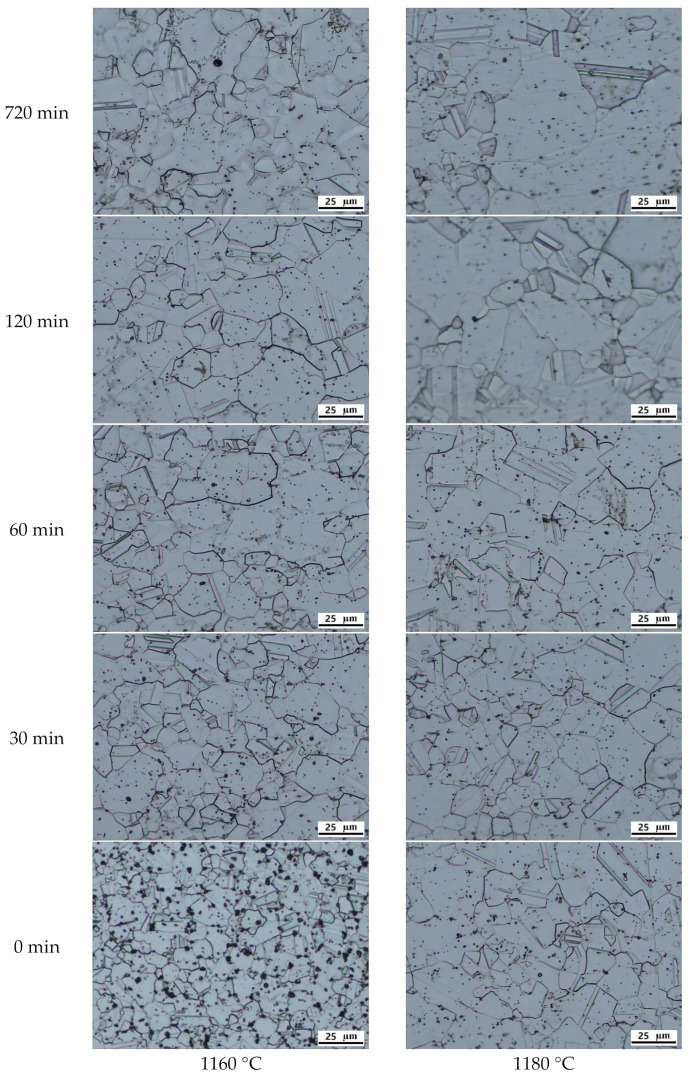
Microstructure evolution at different temperatures and holding times.

**Figure 6:**
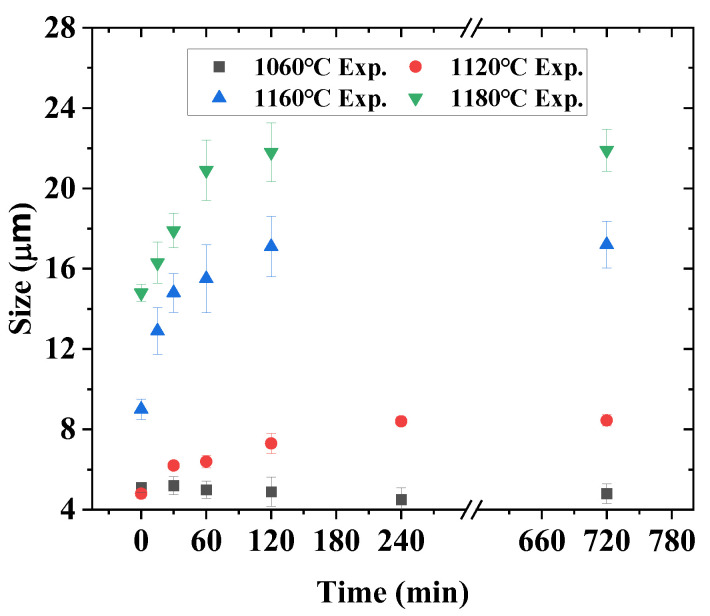
Grain size grows with time.

The initial forged-state microstructure is shown in Figure 4. The grain structure is rather uniform after extrusion and forging. The microstructure evolution after heat treatment at different temperatures and soaking times can be found in Figure 5. The grain size of each test piece was measured and plotted in Figure 6. The measurement error is within a 95% confidence interval. Grains grow relatively slowly when the heat treatment temperature is between 1060 °C and 1120 °C. The grain size increases from 4.8 μm to 8.4 μm only when the temperature is 1120 °C, even after 240 min. The grain size remains unchanged if the temperature is 1060 °C. However, if the temperature is 1160 °C or 1180 °C, the grains grow much faster even after 15 min of holding, as large as 12.9 μm and 16.3 μm, respectively. The growth rate slows down gradually with the increase in holding time. After holding for 2 h, the grain sizes are 17.1 μm and 21.8 μm for those two heat treatment temperatures. Thermodynamic calculation shows that the γ` solves temperature of the alloy is 1150 °C. This is one of the reasons why the grain grows relatively slowly when the heat treatment temperature is lower than 1150 °C. The grain growth is impeded by γ` phase due to the pinning effect. However, when the temperature is higher than 1150 °C, the grains grow rapidly because of the dissolution of the γ`. As also shown in Figure 6, the grain will not grow forever with the increase in holding time. The grain size will reach an equilibrium state for each heat treatment temperature, which is consistent with the previous discussion results.

### 4.2. Parameter Calibration of Grain Growth Model

Since there is no obvious grain size change in the experimental data at 1060 °C, it is believed that the initial grain size exceeds the limit grain size. Therefore, only the experimental data of 1120 °C and 1180 °C are used in the equation parameter calibration. In order to calculate the saturated grain size in Equation (17), parameters a and b need to be determined.

**Figure 7:**
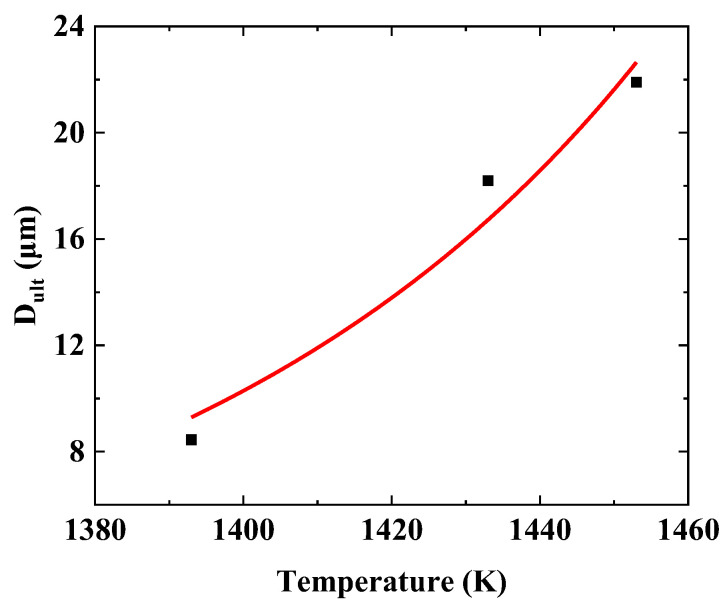
Fitting curve of Equation (17).

In this example, the grain size of samples that experienced 720 min heat treatment was assumed to be the ultimate grain size $D_{ult}$. Equation (17) was fitted in Figure 7 based on the measured grain sizes, and the material parameters are derived a=−114.5628, b=−18.6934, Ts=1598K (assuming to be the melt temperature).

Then Equation (10) can be written as follows:

(18) $$\frac{D^{n}-D_{ult}^{n}}{D_{0}^{n}-D_{ult}^{n}}=\exp\left(-A_{ult}\exp\left(\frac{-Q_{ult}}{RT}\right)t\right),$$

**Figure 8:**
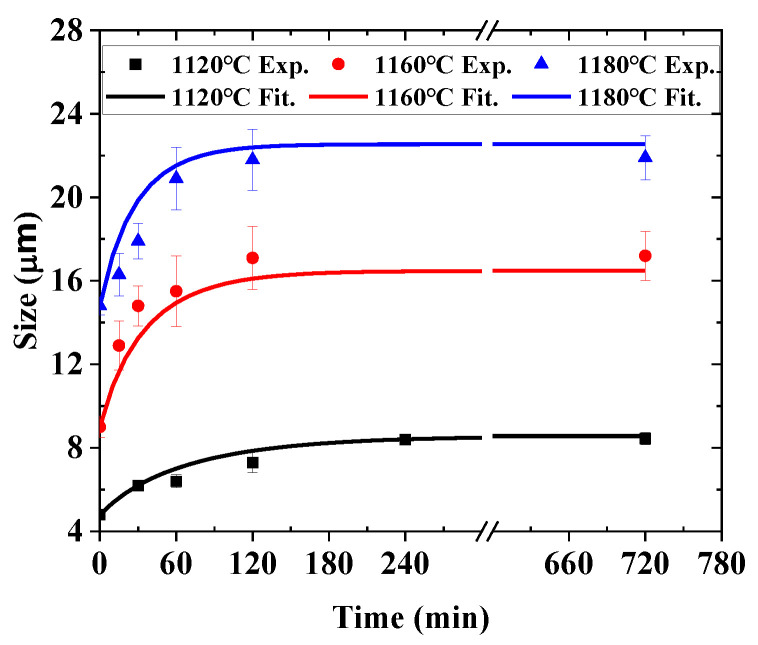
The improved grain growth model prediction by parameter calibration.

**Figure 9:**
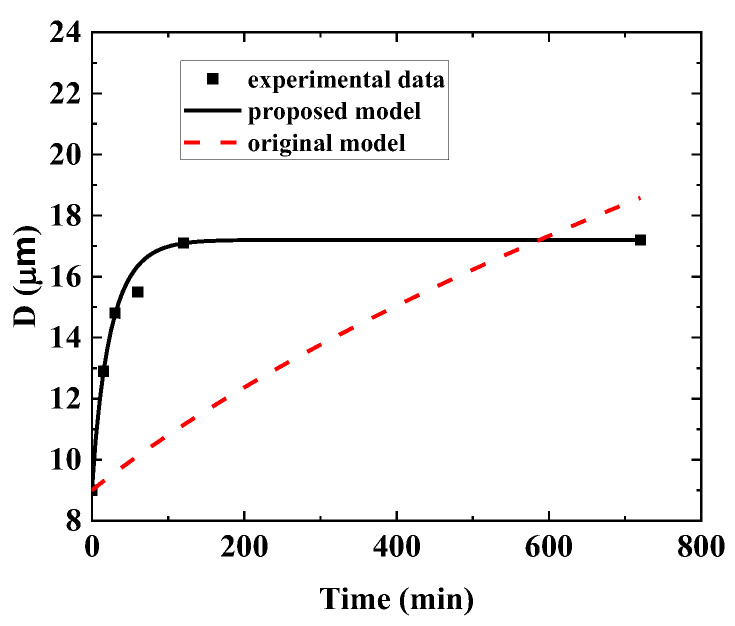
Comparison between the original model and the current proposed model.

After experimental data fitting by Equation (18), the material parameters can be determined as $A_{ult}=2.06547\times 10^{15}$ and $Q_{ult}=5.15704\times 10^{5}$, n=2, and the fitted curves are plotted in Figure 8. The proposed model exhibits an obviously higher accuracy than the original model. Moreover, as an example, the comparison between the original model and the current proposed model is shown in Figure 9. It shows the improvement of the current proposed model.

### 4.3. Grain Size Calculation Scheme

In order to calculate the grain size from temperature evolution, the Scheil superposition method is used to discretize the continuous change of temperature into a discontinuous isothermal process with multiple incremental steps. In step *m*, the discrete isothermal grain growth increment is calculated at the current temperature:

(19) $$∆\left(D^{n}\right)=max\left[(D_{ult}^{n}{-D_{m-1}^{n})A}_{ult}exp(\frac{-Q_{ult}}{RT})∆t,0\right],$$

The grain size at the end of step *m* can be calculated as:

(20) $$D_{m}^{n}=D_{m-1}^{n}+∆\left(D^{n}\right),$$

**Figure 10:**
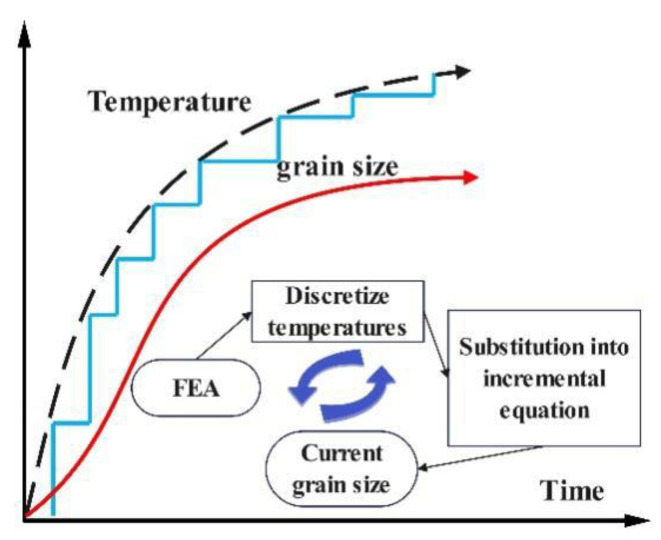
Illustration of the iterative calculation algorithm.

Repeat the operation until the completion of the whole heat treatment process, as shown in Figure 10.

## 5. Grain Size Prediction of a Dual Microstructure Turbine Disk and Its Validation

The turbine disk is one of the critical components of an aero-engine turbine. Its performance directly determines the overall performance of the engine. During the service of a high-performance aero-engine turbine, the temperature at the rim area is high, and the coarse-grained structure is preferable to ensure high durability and creep performance. The operating temperature of the bore region is relatively low but subject to large torsion and centrifugal force, which requires a fine grain structure to provide excellent tensile and fatigue strength. The dual-performance powder disk can maximize the performance potential of the material and better meet the actual working conditions of different parts of the turbine disk [21]. Simulation is the most effective and efficient method to help design the gradient heat treatment scheme by accurately predicting the temperature field of the disk in order to have the right grain size distribution for the dual performance requirement [22,23].

### 5.1. Gradient Heat Treatment Setup Design

In order to let the rim and bore of the disc have different grain sizes, it is needed to form a temperature gradient on a single disc. There are many ways to achieve this [24,25,26,27,28,29]. Among them, only heating the disk edge by adding an endothermic block to the core is easy to implement. The key is how to control the effective heating time and design the size of the thermal insulation device and endothermic block to meet the temperature requirements of each region. The setup and processing parameters can be obtained with the help of an accurate simulation.

**Figure 11:**
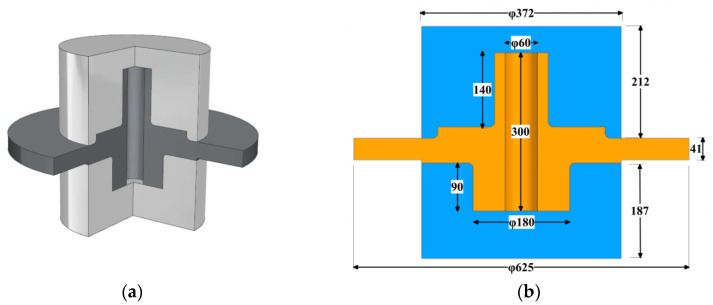

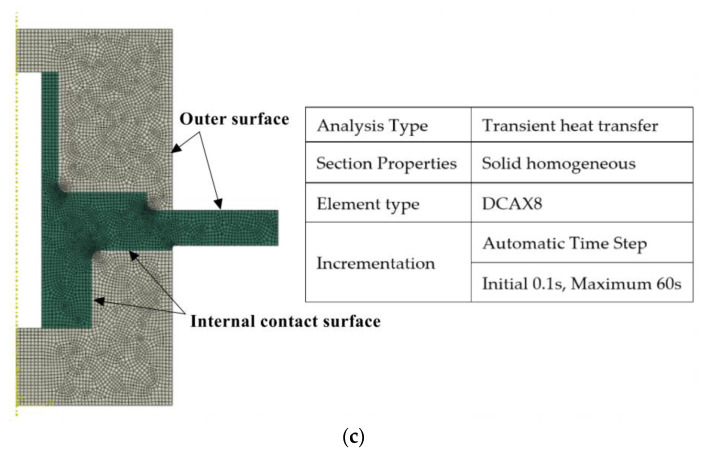
Heat treatment model with thermal insulation. (**a**) 3D model section; (**b**) main dimensions of the model in mm; (**c**) finite element model and boundary parameters.

The setup of the disk heat treatment is presented in Figure 11. The simulation model with tooling is illustrated in Figure 11a. The gray part is the insulation, and the black part is the disc body. Because the disc and shaft are together in this case, there is no need to add additional heat-absorbing blocks, which are normally needed to prevent the temperature from rising at the bore area. And the main dimensions of the overall model are shown in Figure 11b.

The heat transfer can be expressed by the following formula:

(21) $$Q=Flux+h(T-T_{a}\;)+\sigma \varepsilon \left(T^{4}-T_{a}^{4}\;\right),$$

where Flux corresponds to an external flux. $h(T-T_{a}\;)$ corresponds to convective heat transfer between the part surface and the medium, where h is the convective heat transfer coefficient and $T_{a}$ is the environment temperature. $\sigma \varepsilon \left(T^{4}-T_{a}^{4}\;\right)\;$is the radiation heat transfer term. The radiation heat exchange is proportional to the fourth power of the temperature difference between surface and ambient, where σ is the Boltzmann constant and ε is the emissivity. After tedious tuning of parameters by validating predictions against thermal couple measurements, the radiation heat transfer coefficient between the outer surface and the furnace is determined to be 0.5, the convection heat transfer coefficient is 10 W/(m^2^·K), the contact heat transfer coefficient between the disc and the thermal insulation tooling is 50 W/(m^2^·K), and the furnace wall temperature is 1200 °C.

The FEM model was built in ABAQUS 2020. The model adopts quadratic quadrilateral axisymmetric elements (DCAX8). The mesh size is 4 mm. The chamfer is locally densified, and the size is about 1 mm. The specific parameter settings are shown in Figure 11c. The grain size was calculated in the user subroutine UVARM based on the proposed model.

**Figure 12:**
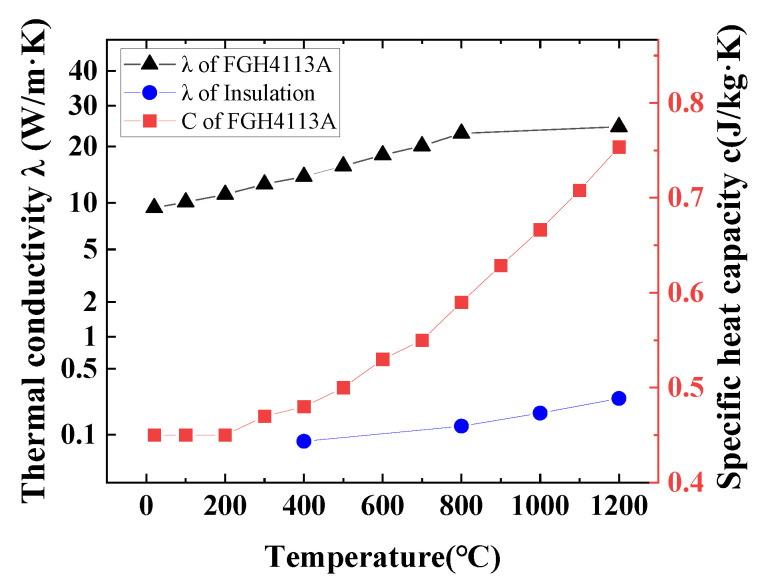
Thermal physical properties of materials in the calculation.

The thermal insulation tooling is made of heat-resistant fiber. The main thermophysical parameters of the materials used in the calculation are shown in Figure 12. The density of FGH4113A is 8300 kg/m^3^. The density of thermal insulation material is assumed to be constant at 450 kg/m^3^, and the specific heat capacity is 0.5 kJ/(kg K).

### 5.2. Heating Process of Gradient Heat Treatment

**Figure 13:**
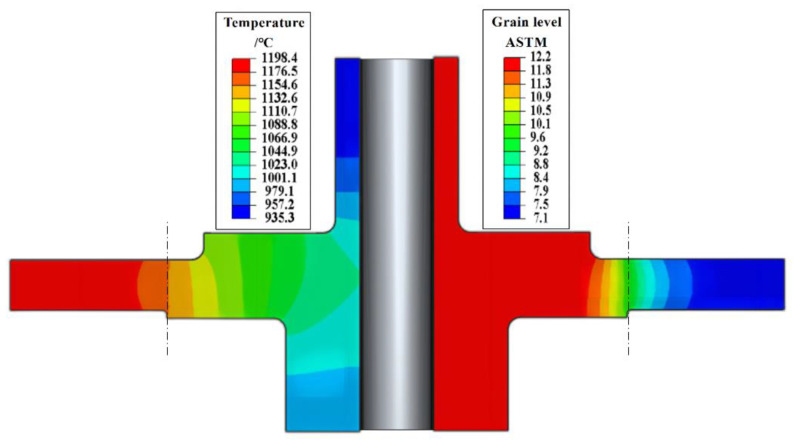
Predicted temperature and grain level distribution of the disk.

In order to obtain the maximum temperature gradient, hot loading is usually used. When the furnace temperature reaches 1200 °C, the whole setup (disc and insulation) is loaded into the furnace. The temperature field distribution is shown in Figure 13 after holding for about 2 h and 55 min. The dotted line position is the boundary of the thermal insulation tooling. It can be found that the temperature on the outside of the dotted line is above 1150 °C, which is higher than the solvus temperature of the material, while the temperature on the inside of the dotted line is lower than the solvus temperature of the material, which is shown on the left part of Figure 13. As a consequence, γ` phase on the rim area will be dissolved so that the grains can grow. While the temperature in the core area is low, only the intracrystalline γ` phase will dissolve, and the grain size will remain basically unchanged, as shown on the right part of Figure 13 by the current model prediction.

Due to the high solution temperature on the rim, the grains grow rapidly, and the grain size is at the level of ASTM 7-8, while the grain size of the bore part is at the level of ASTM 11-12, which is basically maintained at the level of the initial forged state grain size. The transition region is small and rather smooth near the thermal insulation tooling position. The dual microstructure forms dual properties for the disc, which will better meet the operation requirements. Such results also align well with some advanced turbine discs manufactured by those well-known aero-engine OEMs. For example, General Electric Aviation Engine has manufactured a dual-performance turbine disk made of René 104 alloy. The report [30] shows that the grain size levels of its rim are ASTM 6-7, the grain size grade of its hub is about ASTM 11, and the transition zone is ASTM 8-10. NASA’s Glenn Research Center has also manufactured a dual-energy turbine disk of ME209 alloy by a low-cost method [31]. The grain size of the wheel hub is ASTM 11-12, and the rim is about ASTM 5.

### 5.3. Prediction Validation

**Figure 14:**
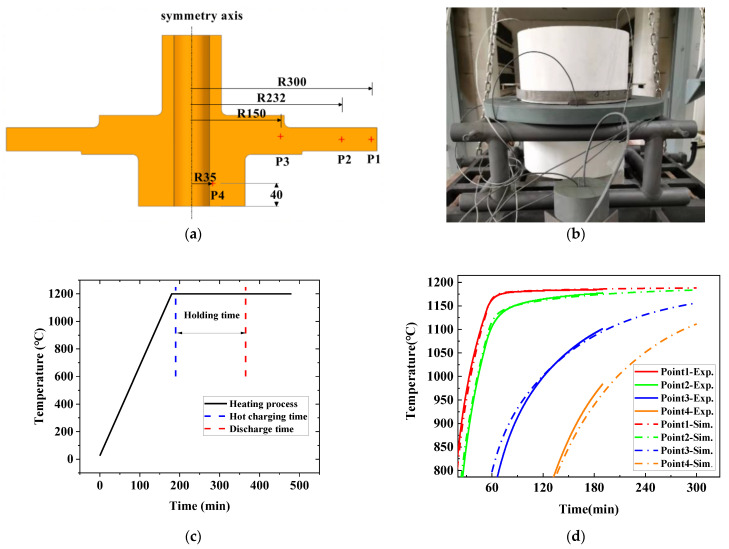
Experimental validation.(**a**) layout of measuring points; (**b**) experimental setup; (**c**) heating process; (**d**) comparison on temperature between experiment and calculation.

Temperature measurement was carried out in order to validate the prediction as well as the effectiveness of the design based on the prediction. The thermal couples were inserted at the points shown in Figure 14a (P1-P2-P3-P4), where P1, P2, and P3 are 1/2 of the thickness and P4 is 40 mm from the bottom.

The setup picture is shown in Figure 14b. The heating equipment is a trolley furnace, and the N-type thermocouples are inserted at the position shown in Figure 14a. The outline dimensions of the powder turbine disk and thermal insulation tooling are already shown in Figure 11b. The furnace is heated based on the curve in Figure 14c. When the furnace temperature reaches the predetermined temperature of 1200 °C, the setup shown in Figure 12b is loaded. After holding another 2 h and 55 min, the setup was discharged. Thermal couples recorded the thermal history throughout the entire process. The whole experimental scheme is shown in Table 3. The same practice had been repeated several times. The results show excellent repeatability for such an operation.

The comparison of temperature variation between simulation and measurement is shown in Figure 14c, while the solid lines are from measurement and the dotted lines are from prediction. The calculated temperature field agrees very well with the measurement through the whole temperature history for all the positions studied. The relative error between the calculation and measurements at the discharging moment is shown in Table 4.

**Figure 15:**
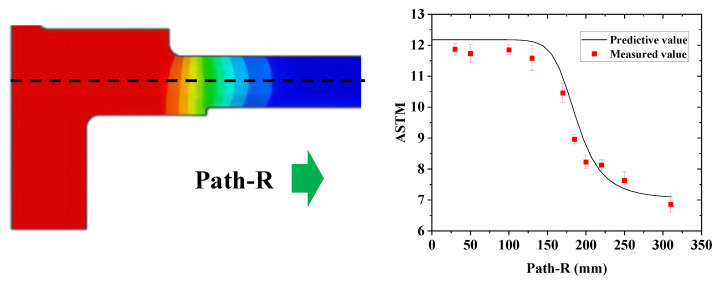
Predicted and measured grain size along the path.

**Figure 16:**
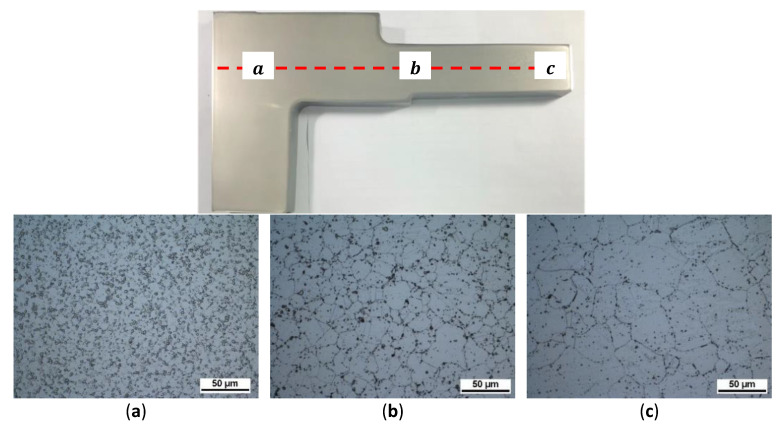
Metallographic structure of different positions. (**a**) position a at the bore; (**b**) position b at the transition zone; (**c**) position c at edge of the plate.

After dissecting the disc, a metallographic examination was performed. The grain size was measured. The comparison results between prediction and measurement are shown in Figure 15. The path in the radial direction is defined, and its horizontal position is half the thickness of the disc edge. The prediction agrees very well with the measurement from bore to rim as well as the transition zone. The metallographic structures at positions of a, b, and c are shown in Figure 16. Position a is located in the bore with fine grains. Due to the low temperature during heat treatment, the grain size changes slightly. However, position c is located at the edge of the plate, and its grain size is coarse due to the sup-solvus temperature it went through. Position b is located at the edge of the heat shield, which is the transition zone, and its grain size is in between. The grain sizes of the three areas are ASTM 11.8, ASTM 9.0, and ASTM 6.8, respectively, which match well with the predicted values of ASTM 12.1, ASTM 9.1, and ASTM 7.1. The comparison results are shown in Table 5.

## 6. Conclusions

In this paper, a grain growth model that considers the Zener pining effect was developed. It was validated by a full-size turbine disc after gradient heat treatment. The following conclusions can be drawn:

(1) A new grain growth model has been developed by combining the traditional model and the Zener pinning effect. This model can reflect how the ultimate grain size influences grain growth.
(2) A simplified temperature-dependent ultimate grain size model has been proposed. It can be applied flexibly to materials exhibiting both single and multiple pinning mechanisms.
(3) Isothermal grain growth experiments were conducted to study the grain growth of a nickel-based superalloy and to validate the proposed model.
(4) The proposed grain growth model with a saturated grain size term can better predict grain growth. The model parameters were calibrated by experiments on small samples.
(5) The proposed model was applied to a finite element analysis of the gradient heat treatment of a full-size turbine disc. The predicted grain size distribution matched the results of the metallographic examination very well. It demonstrates that the model can offer valuable guidance for optimizing subsequent processes to meet different requirements. This method can be extended to other alloys.

## Figures and Tables

**Table 1:** Nominal composition of FGH4113A alloy (wt.%).

Co	Cr	Al	Ti	Ta	W	Mo	Nb	C, Hf, B, Zr	Ni
19	13	3.0	3.7	1.0	4.0	4.0	1.2	Minor	Bal.

**Table 2:** Isothermal experiment design.

Temperature (°C)	Soaking Time (Minutes)					
1060	0	30	60	120	240	720
1120	0	30	60	120	240	720
1160	0	15	30	60	120	720
1180	0	15	30	60	120	720

**Table 3:** Experiment arrangement.

Steps:	Details:
Experimental preparation	1. Drill temperature measuring holes according to the requirements of measuring points 2. Place the turbine disk and assemble the heat-insulating tooling 3. Fix the thermocouple and connect the temperature recorder
Experimental equipment	Trolley furnace
Charging mode	Hot charging
Heating process	As shown in Figure 14c

**Table 4:** Comparison of temperature between prediction and measurement.

Location	Prediction /°C	Measurement /°C	Relative Error /%
Point 1	1186	1185	0.1
Point 2	1174	1178	−0.3
Point 3	1095	1101	−0.5
Point 4	964	984	−2.0

**Table 5:** Comparison of grain size level at measuring positions.

Measuring Point	Calculated/ASTM	Measured /ASTM	Relative Error /%
Position *a*	12.1	11.8	2.0
Position *b*	9.1	9.0	1.5
Position *c*	7.1	6.8	5.0

## Data Availability

Not applicable.

## Abbreviations

a	material parameters
A	the generalized mobility constant
$A_{ult}$	the generalized mobility constant considering ultimate size
b	material parameters
C	Zenner pinning force constant
D	grain size
$D_{0}$	initial grain size
$D_{ult}$	the ultimate size
f	the volume fraction
K	temperature-dependent constant
$K_{ult}$	temperature-dependent constant considering ultimate size
n	time-dependent exponent
Q	grain growth activation energy
$Q_{ult}$	grain growth activation energy considering ultimate size
r	radius of undissolved and coarsened particles
R	gas constant
t	annealing time
T	annealing temperature
$T_{s}$	solvus temperature of the pinning phase
γ	the grain boundary energy
AA	argon atomization powder production
ASTM	grain size level
FEM	finite element method
HIP	hot isostatic pressing
HEX	hot extrusion
IF	isothermal forging
VIM	vacuum induction melting
UVARM	user-defined state variables
